# Combining technical and expert-by-experience knowledge in the quest for personal recovery from bipolar disorder: a qualitative study

**DOI:** 10.1186/s12888-019-2357-3

**Published:** 2019-11-26

**Authors:** Samson Tse, Winnie W. Y. Yuen, Greg Murray, Larry Davidson, Queenie Lai, Alice Kan

**Affiliations:** 1 Department of Social Work and Social Administration, The University of Hong Kong, Hong Kong, China; 2 Current address: Department of Counselling and Psychology, Shue Yan University, Hong Kong, China; 3 Department of Psychological Sciences, Swinburne University of Technology, Melbourne, Australia; 4 Yale Program for Recovery and Community Health, Yale University, New Haven, USA

**Keywords:** Bipolar disorder, Health communication, Knowledge transfer, Mood disorders, Mental illness, Peer support service, Recovery

## Abstract

**Background:**

Knowledge construction is a form of communication in which people can work individually or collaboratively. Peer support services have been adopted by the public psychiatric and social welfare service as a regular form of intervention since 2015 in Hong Kong. Peer-based services can help people with bipolar disorder (BD) deal with the implications of the diagnosis, the way in which individuals with BD receive treatment, and the lifestyle changes that take place as a result of the diagnosis. Through a qualitative paradigm, this study aims to examine how individuals with BD use technical and expert-by-experience knowledge.

**Methods:**

A total of 32 clients of mental health services were recruited from hospitals, Integrated Community Centers for Mental Wellness, and non-governmental organizations. They participated in semi-structured individual interviews. All interviews were recorded, transcribed verbatim, and analyzed using thematic analysis with the aid of NVivo. The findings were verified by peer researchers.

**Results:**

Three main themes are presented in this article, including how clients made sense of the knowledge provided by mental health professionals and peer support workers (PSWs), critical perspectives about peer support services, and the way in which the services are more than knowledge transfer alone. Participants generally indicated that knowledge sharing revolved around three experiences: mood changes, medications, and sense of hope. Nevertheless, an empathic understanding of the clients’ experience was more important than the sharing of knowledge. Some clients perceived medication as the chief means to recovery, so PSWs were not useful for them. However, PSW role models had an effect beyond mere knowledge transmission, as they could promote clients’ pursuit of functional recovery goals.

**Conclusions:**

The present study has improved our understanding of knowledge sharing between clients with BD and health professionals or PSWs, which should take place in an empathic and hope-instilling manner. It has also emphasized the value of the presence of a role model who can speak convincingly with clients to facilitate recovery. The present findings can be used to improve the care of people with BD by generating important guidance with regard to enhancing the knowledge exchange between clients and health practitioners.

## Background

Bipolar disorder (BD) is a chronic mood disorder characterized by striking and persistent swings between two extreme phases: 1) the manic phase, characterized by a sustained high mood and impaired judgment (e.g., overspending, hyper-sexuality); and 2) the depressive phase, which is often accompanied by self-harm behaviors. According to the World Health Organization’s World Mental Health Survey Initiative, about 74% of those with bipolar depression and half of those with bipolar mania report severe impairment in their various life roles, and 16 and 21% of those with BD respectively, will attempt suicide or have suicidal plans in their lifetime [1]. BD is a condition with significant medical, interpersonal, and economic consequences. It affects 2% of the adult population in the US, and subclinical conditions affect another 2.4%. The annual cost of BD in the US has been estimated at US$45.2 billion, with 70% of this being attributed to lost productivity [2, 3]. The 12-month point prevalence rate of BD in Hong Kong is estimated to be between 0.5 and 1.8%, affecting between 36,000 and 129,000 individuals. While medication remains the first-line treatment for the acute mania of BD [4], different forms of psychosocial intervention exist for further functional improvement, such as cognitive behavioral therapy, interpersonal and social rhythm therapy, family-focused therapy, and psychoeducation [5].

### Recovery approach and peer support service

Trends in healthcare and research regarding the treatment of severe mental illness are shifting. Professionals are paying more attention to the experience of living with the condition and to personal recovery. “Personal recovery” refers to the process of individual psychological adaptation to a disorder rather than a sole focus on the reduction of psychiatric symptoms and functional deficits [6, 7]. A study in the UK looked at the meaning and process of recovery through in-depth interviews with 12 people with BD [8]. The key theme that emerged was that recovery is not simply about the absence of symptoms; it requires one to make sense of knowledge from multiple sources and to take responsibility for one’s own wellness [9–11]. Within such a recovery approach, peer support services have been emerging and mental health services have been progressing toward being recovery oriented. The ideas behind peer support service grew out of the service user or survivor movement and thus outside of the traditional mental health arena [12, 13]. The service draws on the recovery values of hope, self-determination, participation in self-care, and use of knowledge gained from lived experience to help others [14, 15]. The basic qualities of peer support service include the following three aspects: 1) instilling hope through positive self-disclosure; 2) the role modeling of self-care in relation to the illness and “exploring new ways of using experiential knowledge” in managing day-to-day life [14, 16]; and 3) relationships between PSWs and clients that are characterized by “trust, acceptance, understanding, [and] the use of empathy, empathy which in this case is paired with conditional regard” [14].

For instance, a qualitative one-year longitudinal study in Hong Kong found peer support services to improve motivation, positive thinking, empowerment, physical well-being, and connections with other clients [17]. Several effectiveness studies and reviews have shown that peer support services are able to instill a sense of hope, control, and self-efficacy; to enhance self-care, relationships with service providers, and participation in treatment; to increase a sense of belonging to a community and satisfaction with life; and to reduce psychotic and mood symptoms among clients with mental illness [18–23]. Recent studies on peer support services focus on three main areas: 1) conducting high-quality clinical trials [14, 17, 20, 23, 24]; 2) studying what works to achieve an organizational transformation or cultural workplace shift to better implement peer support services [14, 23, 25, 26]; and 3) deepening our understanding of the mechanism of how PSWs work [14, 21, 27, 28]. One of the mechanism-related questions is as follows: How do clients conceptualize the knowledge derived from mental health professionals and PSWs to manage their BD? For instance, Asian and Chinese people in particular tend to assume that authoritative knowledge is distributed hierarchically, and they give greater credibility to information offered by professionals, sometimes to the exclusion of all other sources [29, 30]. Furthermore, a recent Hong Kong study found that professional staff tend to adopt an authoritarian and pessimistic view of what PSWs can contribute to mental healthcare [31].

### Knowledge acquisition to support recovery

To the best of the authors’ knowledge, this is the first study to examine the services provided by PSWs from the perspective of knowledge acquisition. Knowledge is acquired by people working individually or collaboratively. It may be generated through reading, formal education, upbringing, or working relationships, and is often bounded by what society believes to be legitimate [32, 33]. Knowledge construction (e.g., health concepts and disease coping strategies) and communication are subject to human vices and virtues [32]. Knowledge is “never a neutral or objective phenomenon but a matter of positionality, that is, of the place from which one speaks, to whom and for what purpose” [34]. How can clients—who are often considered by society as “insane and irrational”— and mental health professionals with statutory power work together to co-construct knowledge? What is transferred is not “the sum of what is known” by the people imparting it, but an imperfect and incomplete subset of their knowledge [33]. The starting point for a sociological analysis of medical versus lay knowledge of healthcare dates back to the early 1970s, with Freidson’s landmark publication [35]. Medical (technical) knowledge primarily concerns diseases and treatments; lay knowledge rests upon personal experience of illness [36]. Table 1 compares the features of the two forms of knowledge: technical knowledge and expert-by-experience knowledge. “Expert-by-experience knowledge” was first used in a reflexive-collaborative study exploring recovery from BD [37]. We use this term to refer to the experiential knowledge passed on by PSWs [10]. The position adopted in the present study is that both forms of knowledge are valid and that knowledge is most powerful when the two are combined under terms of mutual understanding, respect, and equality.

**Table 1:** Continuum of technical knowledge and expert-by-experience knowledge

	Technical knowledge	Expert-by-experience knowledge
Features	• Scientific • Systematic and well-documented • Common languages shared across multiple professionals • Findings are not always relevant to service users’ lived experience • It greatly depends on practitioners’ ability to understand a perspective other than their own and to respond empathetically	• Acquired through experience: “been there, done that” • High ecological validity, very practical • It does not always generalize to other people’s circumstances • It depends on the service setting, training, and skills of PSWs, such as interpersonal skills, adjustment to the new PSWs role
Challenges	• Combining technical and expert-by-experience knowledge in the search for personal recovery is not always a straight-forward process. The two forms of knowledge sometimes work in a complementary manner, but at other times they work in a more tensioned, question-raising way that can broaden our understanding of how knowledge is interpreted by multiple parties (e.g., mental health practitioners, clients or family members) • There is a pressing need to move from a situation in which health knowledge construction is hierarchical to one in which it occurs by consensus, horizontally. Such a shift would allow healthcare professionals and clients to contribute to the co-construction of knowledge that forms the basis for decision-making in the recovery journey.	

### Peer support services and bipolar illness

Peer support services are of particular importance to people newly diagnosed with BD. Research indicates that most people with BD struggle to accept all the implications of the diagnosis for a period of time because they find it difficult to receive long-term treatment and to make long-lasting lifestyle changes [38]. The first step to self-managing the illness is acceptance of the diagnosis [9]. It has also been shown that most people with BD face specific barriers to practicing self-management of their illness, such as a lack of communication with service providers and insufficient access to treatment, which may be tackled by providing social and peer support service [10, 39].

Accumulating research evidence also indicates that BD is linked to more advanced cognitive functions, as reflected in excellent pre-morbid school performance [40, 41] and superior leadership qualities [42]. As such, people with BD tend to have a high demand for rationalization of their illness and for a thorough understanding of their condition. In order to unleash their potential in education and employment, they also need to come to terms with the illness in order to attain better self-management. A study using peers as “informed peer supporters” in psychoeducation showed that they brought several benefits to individuals newly diagnosed with BD, including the provision of emotional support, practical strategies for illness management, the encouragement of positive relationships with mental health services, and the provision of a role model for treatment adherence [28]. The studies reviewed above mainly come from Western countries; studies focusing on the experiences of those receiving peer support service in non-Western regions, such as Hong Kong, Taiwan, and Singapore, or lower-middle-income and low-income countries (e.g., India, Uganda, and Tanzania) are in early stages [12, 43, 44]. It is uncertain how expert-by-experience knowledge is viewed in Chinese culture and how PSWs are perceived in the existing mental health system.

The key milestones of the development of peer support services in Hong Kong are summarized as follows. The most recent Hospital Authority estimates suggest that up to 1.7 (out of 7.3) million Hong Kong people suffer from different types of mental illnesses, with 70,000 to 200,000 of them considered to have severe conditions. The first pilot peer support training in Hong Kong was conducted by Caritas social services, funded by The Community Chest of Hong Kong; it ran between 2011 and 2014 [12]. Another major territory-wide initiative was conducted under the auspices of the Peer Support Workers Project and jointly implemented by four non-governmental organizations (NGOs). The project has three components: A pre-service training program on the provision of peer support services and interpersonal skills delivered in 16 three-hour sessions; a 52-h work placement; and paid employment [17]. A total of 60 people with lived experience of mental health conditions completed the training and 12 full-time equivalent PSW posts for 12 months were created. This three-year (2012–2015), multi-organizational project was supported by MINDSET, a philanthropic initiative established by the Jardine Matheson Groups, Hong Kong. The turning point came about in 2015 when the immediate past Chief Executive announced in his Policy Speech that the Hospital Authority, which runs the three main regional psychiatric hospitals, would strengthen the manpower of the psychiatric healthcare team and introduce peer support services to the case management program for clients with severe mental illnesses. Subsequently, the Social Welfare Department implemented the two-year pilot project on peer support services in community psychiatric service units in March 2016. Within the public hospital sector, as of April 2019, a total of approximately 20 full-time equivalent PSWs had been recruited. The peer support service provided in the social welfare sector has been established as formal and regular intervention since March 2018, with the number of full-time and part-time PSW positions increased to about 50.

### Study aim and research questions

The overall aim of this study is to examine how clients with BD seek, assess, and use technical and expert-by-experience knowledge (provided by PSWs based on their own lived experience of mental illness) to support their recovery process. How do people who are learning to live a meaningful life despite BD make sense of the knowledge provided by mental health professionals and PSWs? What are the differences in how clients at different recovery stages (e.g., initial versus late stages) make sense of knowledge from mental health professionals and PSWs? Are there any unintended consequences of the two types of knowledge? The present study tackles the above questions via qualitative interviews with clients of peer support services in different settings who have been diagnosed with BD in Hong Kong.

## Method

### Design

The study adopted a qualitative paradigm underpinned by an interpretive approach representing a social constructionist view of reality [45, 46]. Data were collected from 32 clients with BD via individual interviews. Our earlier study revealed that the strongest predictor of recovery for adults with BD in clinical remission is “respect, hope, and empowerment” [47]. This finding explains why we focused on BD to examine how clients are empowered to use technical and expert-by-experience knowledge to support their recovery.

### Participants and setting

Participants were identified by purposive sampling in hospitals, Integrated Community Centres for Mental Wellness (ICCMWs), and NGOs for people with mental illnesses, in order to capture people’s experiences of using peer support services in different settings. The inclusion criteria required individuals to be aged 18 or above, ethnically Chinese, diagnosed with BD by a psychiatrist, currently receiving treatment from a mental health professional and formal peer support services, able to participate in a 45-min interview, and able to give written informed consent. After the purpose of the study and their rights were explained, written consent was obtained from all participants. A total of 32 clients were recruited from a hospital (*n* = 8), ICCMWs (*n* = 14), and NGOs (*n* = 10) (Table 2). Data collection was discontinued when no new codes occurred in the data and saturation was achieved. On average, the participants had lived with BD for 21 years (range = 5 to 37 years), 71.9% were female, and their mean age was 46.3 years (range = 26 to 66 years). The peer support services they received varied in terms of setting; they included one-to-one conversations with PSWs (on phone or in person) and joining group activities accompanied by PSWs or run by PSWs (Table 2). The participants received a supermarket coupon worth USD12.50 as a token of the authors’ appreciation. The Institutional Review Board of the University of Hong Kong/Hospital Authority Hong Kong West Cluster approved this study.

**Table 2:** Characteristics of the study sample

Participant number	Gender	Age	Year of diagnosis	Settings^a^ where the participants were recruited	Main types of peer support services received^b^ (Services provided by general mental health practitioners^c^)
SU01	F	55	20	ICCMW	Recovery group led /co-led by PSW
SU02	F	48	30	ICCMW	Recovery group led /co-led by PSW
SU03	M	52	16	In-patient Hospital	One-to-one in person conversation
SU04	M	26	10	ICCMW	Phone conversation
SU05	F	42	16	ICCMW	Phone conversation
SU06	F	58	31	In-patient Hospital	Recovery group led /co-led by PSW
SU07	F	40	18	Community	Group sharing
SU08	F	59	33	Community	Group sharing
SU09	M	39	20	In-patient Hospital	One-to-one in person conversation
SU10	F	62	26	In-patient Hospital	Group sharing
SU11	F	26	8	In-patient Hospital	One-to-one in person conversation
SU12	M	36	24	Community	Group sharing
SU13	M	63	36	Community	Group sharing
SU14	F	54	37	Community	One-to-one in person conversation
SU15	F	53	15	Community	One-to-one in person conversation
SU16	M	46	30	Community	Group sharing
SU17	F	49	16	Community	One-to-one in person conversation
SU18	M	45	28	Community	Group sharing
SU19	F	42	18	Community	Group sharing
SU20	M	27	5	In-patient Hospital	One-to-one in person conversation; Phone conversation
SU21	F	48	29	In-patient Hospital	One-to-one in person conversation; Recovery group led/co-led by PSW
SU22	F	47	15	ICCMW	Phone conversation
SU23	F	54	19	ICCMW	Leisure group
SU24	M	66	20	ICCMW	One-to-one in person conversation
SU25	F	27	12	ICCMW	One-to-one in person conversation
SU26	F	52	24	ICCMW	Recovery group led /co-led by PSW
SU27	F	53	37	ICCMW	Recovery group led /co-led by PSW
SU28	F	28	9	In-patient Hospital	One-to-one in person conversation
SU29	F	58	15	ICCMW	One-to-one in person conversation
SU30	F	40	13	ICCMW	Recovery group led /co-led by PSW
SU31	F	44	26	ICCMW	Leisure group
SU32	F	41	17	ICCMW	Recovery group led /co-led by PSW

Notes.

^a^Settings: *ICCMW* Integrated Community Centre for Mental Wellness. Community settings include faith groups, general social groups.

^b^Main types of peer support services: Recovery group covers various topics e.g., goal setting, meaning of “personal recovery”. One-to-one in person conversation refers to the participants talking with PSW about various topics e.g., the activities the participants are doing, personal matters concerning them. Phone conversation refers to the participants who are using the agency’s warm line (phone-based mutual care service). Examples of leisure group are cooking sessions, Tai-Chi class, praise and dance group.

^c^Services provided by general mental health practitioners: The participants received a variety of mental health services offered by the general mental health practitioners, e.g., psychiatric services in the hospital or at the specialist out-patient clinics, community psychiatric nursing services, occupational therapy, case management by social workers at the ICCMW, and psychotherapy or counselling (which were rarely mentioned by the participants in the present study).

### Data collection

Two of the authors (ST and WY) developed an interview guide with open-ended questions based on the literature on BD, recovery, and the research aims. The questions covered areas including the mental health services the participants had been using, their understanding of recovery and their recovery journey, and their perception of PSWs and the professionals aiding their recovery. Sample questions include: “What does ‘recovery’ mean to you?”, “Describe your experience with mental health professional/PSWs”, and “What are the differences and similarities between the knowledge (e.g., information about the condition, wellness management methods) provided by mental health professionals and PSWs?” An interview guide was previewed by two peer researchers with lived experience of BD and were modified based on their feedback; for example, regarding who they would turn to when feeling unwell. All interviews were conducted in Cantonese by one person (WY) in a quiet room at the place where the participants were receiving services. The interviews lasted between 30 and 65 min. Audio recordings were made and these were then transcribed verbatim in Chinese by professional transcribers and saved in encrypted files for subsequent analysis and interpretation. All personally identifiable information was removed from the texts to ensure confidentiality and quality checks were performed by WY, by comparing the recordings to the transcripts. Only the direct quotations cited in the present manuscript were translated into English.

### Procedure and research rigor

Thematic analysis was performed using the NVivo 11 qualitative data analysis software. During the data collection stage, WY and ST met regularly to review the findings and to explore the deeper meanings of recurrent information that might lead to new emergent themes or the necessity to ask additional questions. Following Braun and Clark’s six-phase thematic analysis guide [48], QL and ST read all of the interview transcripts independently to familiarize themselves with the data. They then met several times to discuss the initial codes, while remaining open to new codes generated from the data. The interviews were coded using the agreed system and potential themes were identified and reviewed, resulting in three overarching thematic components. Rigor was achieved by two methods. First, the results were reviewed and verified by the two named investigators who have lived experience of bipolar illness; second, the findings were presented in a meeting attended by about 50 PSWs and mental health professionals, to seek their input about the interpretation of the findings. The major feedback received was that the unique features of knowledge and how they were shared by PSWs should be presented distinctively.

## Results

The data collected from 32 people with lived experience of BD were voluminous. In this article, we will focus on three main themes: 1) how clients make sense of the knowledge provided by mental health professionals and PSWs; 2) critical perspectives on technical and expert-by-experience knowledge, including reservations and unintended consequences described by clients; and 3) Peer support service is more than mere knowledge transfer. The themes and sub-themes are summarized in Table 3.

**Table 3:** Summary of the findings

Themes	Sub-themes
1. Making sense of the knowledge provided by mental health professionals and PSWs	1.1 Empathic understanding is more important than sharing knowledge. 1.2 Knowledge about mood change, medication, and remaining hopeful.
2. Critical perspectives on technical and expert-by-experience knowledge	2.1 When the role of PSWs was not clear or the PSWs were inexperienced, expert-by-experience knowledge was less helpful. 2.2 Technical and expert-by-experience knowledge were different but somewhat related.
3. It is more than mere knowledge transfer	Role-modeling speaks louder.

### Theme 1: making sense of the knowledge provided by mental health professionals and PSWs

#### Empathic understanding is more important than sharing knowledge

When asked what separated the services rendered by the two groups of workers, one client promptly replied that it was not about the knowledge but the level of empathy demonstrated by PSWs and mental health professionals:

*The most direct thing is that the PSWs could feel how I feel; that is, they resonated with what was in my mind, and empathized […]. The PSWs knew exactly what I was talking about, so I did not have to repeat my history of the last 30 years. (SU21).*

Another client echoed this and added that there was no hierarchy between PSWs and herself, as might be the case in interactions with other professionals:

*They (PSWs) have personally experienced this and knew the entire situation. I shared with the peer worker what I had experienced. We had common ground; we had empathy for each other. We felt that we had the same issue so there was no hierarchy and I was able to speak my mind freely. (SU17).*

#### Knowledge about mood changes, medication, and remaining hopeful

Further analysis of the results revealed that, broadly speaking, clients and health workers shared technical and/or expert-by-experience knowledge regarding three important experiences: mood changes over time, medication, and preserving a sense of hope. Regarding the mood changes associated with BD, some participants gave vivid accounts of conversations of how PSWs were able to understand them. For example, one participant said that:

*The PSW were more able to put themselves in our shoes [than other mental health professionals]. They understood the situation and what was it like to have the illness. For example, it is really uncontrollable when one’s mood is high, which can only be whittled away at by time. That was how I managed the illness. (SU04).*

Other participants shared that PSWs offered knowledge and insights about depressed moods:

*During the depressive phase, I lost my interest in everything, I had a poor appetite, lost a lot of weight, and suffered insomnia. This was severe depression. The peer worker was able to understand when I talked about these symptoms. Some peers might hear voices; I was curious to ask them about this so that I could learn more.* (SU13).

*There are ups and downs. When we are on the downward slope, we should keep going and not give up. The social workers, however, only said, “It’s normal.” They did not understand that the relapse was a source of stress and we might ask ourselves, “Why could I not avoid it?” with feelings of guilt and self-blame. PSWs would comfort us by saying that there was no need to feel guilty and encourage us to try again.* (SU26).

The second commonly discussed area of technical knowledge concerned medication, and the participants felt that PSWs understood them at a very personal and profound level, particularly regarding undesirable side effects:

*Because PSWs used to take the medicine, they know about its effects, unlike normal people who do not understand how we suffer. There is a transition period, which is difficult; the medicine is leading you and you lose your motivation. So, when I see that they (PSWs) have overcome the transition period and become peer workers, I know they can help me.* (SU23).

*Doctors and nurses do not have that keenly felt pain and understanding. They may know it rationally, but they have not experienced it. If they have the experience, they are not doctors or nurses but our peers. Peers really are going through the same thing as me, like taking medication.* (SU08).

On the other hand, some participants described how psychiatrists explained different types of mental illness, onset, and medications used for different conditions (e.g., SU09, SU10, SU16). One participant recalled a vivid conversation she had with her psychiatrist about medication:

*The doctor told me that I am affected by an endocrine imbalance and I need more serotonin, so it’s inevitable for me to take medication. Moreover, I have been admitted to hospital 10 times, resulting in increasing dosages (and my brain has shrunk). Thus, the golden time for me to recover has gone. If recovery means taking no medication, then it is almost impossible in my case. The doctor told me to “treat it as if you are taking vitamin supplements.” I accepted it because I have been taking them for years.* (SU08).

Participants also discussed the issue of not taking medication and gaining a better understanding of how mood stabilizers worked from psychiatrists:

*I secretly stopped taking my medication for one or 2 months. Later, I realized that it was not going to work because my head was muddled. Therefore, I had to be honest with my doctor: “Please do not tell me off; I have not been taking my medication for almost two months. I was fine at the beginning but it has gotten worse.” The doctor then explained to me that there was still medicine in my system for the first few weeks after I stopped the medication, the amount then dropped, and my symptoms came back. The doctor was right, but I did not want to be led by medications for the rest of my life. I now understand that medicines help stabilize my mood. This was an open discussion with my doctor about medication.* (SU23).

The third commonly discussed topic was about remaining hopeful. One of the challenges faced by people living with BD is how to (re) gain a sense of hope when BD is known to be a lifelong condition with reoccurring changes in mood and behavior, resulting in significant difficulties in life, such as in regard to employment, interpersonal relationships, and heavy financial burdens, for example, due to overspending under the influence of elevated mood in some cases. The following interview excerpt summarizes well how keen the client was to rekindle hope as a result of the PSW’s sharing of expert-by-experience knowledge:

*The presence of hope. Let people in recovery know that they can work again, to help others, with hope*. (SU32).

In another instance, a client with BD (SU23) gained hope of achieving advanced recovery through interacting with PSWs during a cooking interest group:

*We have a cooking class and I met a few peers there. After sharing their stories with us, I realized that mental illness is not terminal; we can recover. The peer workers experienced a lot of ups and downs, but they were able to bounce back, teach us how to cook, and share their experience. I feel good that we, who have mental illnesses, are not hopeless.* (SU23).

In addition to the three topics of mood swings, medications, and hope, participants (e.g., SU23, SU30) also recalled how social workers and nurses shared knowledge with them about services or activities available in their community (e.g., accommodation, employment, disability allowance, hobby groups, and events).

### Theme 2: critical perspectives on technical and expert-by-experience knowledge

#### When the role of PSWs was not clear or the PSWs were inexperienced, expert-by-experience knowledge was less helpful

The clients interviewed in this study indicated that the expert-by-experience knowledge rendered by PSWs was not useful in some circumstances. One client (SU05) stated that she “had no idea about the roles and duties of PSWs” and “did not feel that she [the client] needed their services.” Furthermore, another participant (SU28) felt that, sometimes, the recovery story or knowledge about what facilitates recovery was “repetitive” and “if the sharing of knowledge and experience was shallow, it was not very meaningful”; rather, a “deeper interaction and interpretation of the experience” was preferred.

There were also cases where participants found the interaction with PSWs useful because they represented someone to listen to them or they needed information about different services. It was also reported that more experienced PSWs could offer in-depth analysis and helpful advice:

*It is not necessary to mention the illness all the time; it’s like rubbing salt into the wound, which is painful. I asked the peer worker what to do after being discharged from the ward and we analyzed the situation rather deeply together.* (SU28).

#### Technical and expert-by-experience knowledge were different but somewhat related

In the present study, no participants expressed any concerns or critical views about the knowledge they received from mental health professionals, apart from the means by which that knowledge was shared (see the next theme). Participants generally described health professionals and PSWs as having their own focus in practice and contributing in different ways to their recovery from BD. One participant pointed out that she would get technical advice (e.g., dealing with side-effects, changing medications) from medical professionals, share her concerns with social workers (e.g., a relationship at home), and participate in sharing (e.g., activities she enjoyed) and activities with PSWs. She captured the integrated nature of the two groups of practitioners in a very pictorial way, citing an old Chinese poem:

*Although a peony is beautiful, it needs to be supported by green leaves. Psychiatrists and nurses are peonies, while peer supporters are green leaves. They complement each other to improve my condition.* (SU02).

However, two participants adhered to a hierarchical view of power and knowledge between mental health professionals and PSWs:

*Doctors are professionals, so I follow what they tell me. They must be smart and outstanding to perform that role, so I take my prescriptions without questioning them.* (SU29).

*Of course, you need to consult your doctor or social workers; you must be cautious when making decisions. Community center members and peer support workers are not professionals. I listen to what they say, but may not follow their advice. I am not stupid.* (SU24).

Another participant mentioned that technical knowledge (represented largely in terms of medication-related material in the present study) contributed to 70% of a person’s overall recovery from BD; expert-by-experience knowledge provided by PSWs contributed another 10–15%. The participants involved in this study did not reveal any unpleasant experiences in terms of receiving conflicting information about their condition or treatments.

### Theme 3: it is more than mere knowledge transfer

The participants provided lively descriptions of how action speaks louder than words, and how role models were more effective than the mere sharing of knowledge. Participant (SU23) talked about how the functional recovery (e.g., getting a paid job) achieved by a PSW could “serve as a goal” to achieve soon. Another participant (SU15) referred to a PSW as a “life-size cardboard cutout standup”:

*If I attend follow-up consultations regularly, I can live my life, such as going to work or learning something new like cooking. I can achieve that. PSWs are role models showing us what could happen in 5or 6 years and set my mind at ease.* (SU15).

The present results show that role modeling served specific purposes. Peer support service was about empowering the clients to set recovery goals [44], such as getting a job after being discharged and exploring other possibilities and means to get well and “live a holistic life” (SU30). Clients (e.g., SU06 and SU25) were also keen to learn from PSWs how to “prevent the reoccurrence of the illness” (SU25), which is one of the core characteristics of BD.

Finally, our data do not show major differences in the above themes and sub-themes among participants with different lengths or stages of experience of illness in terms of how they made sense of the knowledge provided by mental health professionals or PSWs. We examined the data carefully by rereading the original interview transcripts and using the NVivo program. We divided the participants into three groups (less than 10 years, between 11 and 20 years, and more than 20 years), according to the self-reported years since being diagnosed with BD, and created node matrices that allowed us to cross-tabulate coded contents. Each cell in the matrix represents a node containing the content coded at the intersection of the row (sub-themes) and column (years of living with BD). Although no differences were found in relation to the above themes or sub-themes about knowledge acquisition, it was found that: 1) participants who had lived with BD for less than 10 years appreciated that PSWs would see them as a person and not merely as a “mentally ill patient”; and 2) participants with more than 10 years of experience with BD reported that communication with mental health professionals often concerned routine “stuff” (e.g., how they were sleeping, taking medication, their mood). The NVivo nodes of themes and sub-themes and trails of analyses are available upon request.

## Discussion

The value of this study is that, to the best of the authors’ knowledge, it is the first study to examine, from the first-person perspective of clients diagnosed with BD, the roles played by PSWs and regular health professionals in contributing to recovery from BD in a non-Western context. The impact of the study also lies in its potential to advance our understanding of helpful knowledge, as interpreted by PSWs and professionals in mental healthcare settings. There has been a gradual emergence of lived experience or expert-by-experience knowledge in psychiatry across the globe in the last two decades [49–51]. Yet, there has been virtually no effort to develop strategies for integrating technical and expert-by-experience knowledge. At the same time, the evidence for peer support services in the mental health arena is increasing accumulatively in countries acknowledged to have advanced psychiatric care, including the US, Australia, and, more recently, Hong Kong [14, 19, 21, 24, 52]. For example, a recent randomized controlled trial conducted in Germany found that participants with severe mental illnesses in one-to-one peer support services showed statistically significant improvements in self-efficacy, compared with the control group [20].

Consistent with our previous study, which showed that the strongest predictor of recovery for adults with BD in clinical remission is “respect, hope, and empowerment” [47], the present study’s findings add that knowledge acquisition or sharing between clients and health professionals or PSWs plays a crucial role in promoting recovery in this population. What surprised us is that what impressed the participants most was not the different types of knowledge (technical or expert-by-experience knowledge conveyed by mental health practitioners and PSWs, respectively), but rather how knowledge was being shared. Knowledge must be shared between clients and health practitioners in an empathic and hope-instilling manner and role models speak convincingly to clients [10, 53]. These three aspects of the findings resonate almost perfectly with earlier studies about the change model for peer support interventions and the mechanism involved in the beneficial impacts of peer support services in mental health services [16, 27]. Based on extensive qualitative case studies exploring the introduction of PSWs into 10 mental health services in voluntary and statutory sectors in England, Gillard et al. (2015) found that:

Building *trusting relationships based on shared lived experience* was the primary mechanism underpinning peer worker interventions […]. We observed two parallel mechanisms flowing from the relationship: *role-modeling* of recovery by peer workers; *engaging service users* with mental health services and the community. In-house evaluations of peer worker initiatives often refer to the important role played by peer workers in demonstrating recovery and promoting *a sense of hope*. [27, italics added].

A recent meta-analysis review reiterated the paramount importance of the crucial quality of empathy in therapeutic relationships [54]. The review confirmed that therapeutic alliances between service practitioners and clients were significantly related to the client’s perceptions of the practitioner’s level of empathy and genuineness. Empathic understanding and how it is manifested between two individuals is heavily culture-bound and mediated by verbal and non-verbal expression [55]. Following a discussion of culture and knowledge acquisition to support recovery from BD, the present study offers mixed findings. While the participants regarded mental health professionals and PSWs as having different roles, as well as different knowledge to support them effectively (see Results: Theme 2, SU2), some participants held a hierarchical view of power and knowledge in regard to mental health professionals and PSWs. Literature reviews found that the barriers to implementing PSWs’ roles in mental health services include misunderstanding and negative attitudes toward PSWs, a lack of interest of clients, a lack of recovery-oriented culture [23, 26], and occupational domain challenges amongst PSWs (e.g., a lack of skills using one’s lived experience, stigma attached to the label of a peer provider) [25]. Perhaps cultural issues in knowledge acquisition are unique to the development of peer support services in Hong Kong and other non-Western countries in general [44]. A recent pilot randomized controlled trial conducted in Tokyo, Japan, found that participants in a PSW-led shared decision-making intervention group reported a significantly more positive view of their relationship with their treating psychiatrists, compared to the control group [24]. Among some participants of the present study, only regular mental health workers were considered as professionals, knowledge was heavily organized in a hierarchal fashion (e.g., medical or nursing professionals hold technical knowledge and pass it to patients; see Table 1) and was not co-constructed, and advice from PSWs was to be treated with caution (see Results: Theme 2, SU24). Moreover, some participants in this study perceived mental health professionals and PSWs as belonging to two separate groups (see Results; Theme 2, SU29) without realizing that some health professionals also have their own lived experience of mental health issues but cannot identify themselves as such for a variety of reasons, such as fear of breaching confidentiality and stigmatization [56]. For instance, it was estimated that 10–20% of doctors become depressed and are at greater risk of suicide or substance misuse than the general population [56, 57]. The proportion of the mental health workforce that has lived experience of mental health issues and how these individuals make use of their first-hand experience (or do not) to contribute to their clients’ recovery remains a heavily under-researched topic.

This study has several limitations. First, we were not successful in recruiting people who were newly diagnosed with BD or new to the healthcare system. It might be that such people were still in the initial stages of coming to terms with their illness and had not started to use peer support services or were not ready to be involved in psychosocial research. Moreover, other clinical characteristics of clients, such as their history of psychotic features and the “dose” of peer support services received (e.g., nature of the services and the frequency of receiving the support) were not available in current study. Future research should include these variables so as to expand our understanding of how peer support works in different subtypes of BD in both Western and non-Western settings. Furthermore, the experiences reported by the research participants about how they used technical and expert-by-experience knowledge were not triangulated by the service providers. Lastly, while this study provides useful findings regarding the application of PSWs in non-Western culture, this may also limit the generalizability of findings to only Asian populations.

Directions for future research include: 1) the design and implementation of a quantitative study to measure the categories of knowledge or information (e.g., scientific, expert-by experience, or service-related knowledge) covered by professionals and PSWs, in order to further investigate the distinctive roles played by mental health professionals, PSWs, and those with a dual background; 2) a longitudinal study of a series of paired case studies (i.e., collecting triangulated data [such as dose of services] from clients with BD [initial and advanced stages of recovery], PSWs, and mental health professionals) over time will shed further light on the topic.

## Conclusion

The present study has improved our understanding of how clients apply the knowledge gained from mental health professionals and PSWs in making healthcare and everyday life decisions. Such an understanding can help service providers to determine effective ways of developing and delivering healthcare information via various clinical encounters. The present findings can be used to generate important guidance (e.g., establishing therapeutic alliances, in-depth sharing of recovery journeys) to enhance the knowledge exchange between clients and health practitioners, in order to improve the care of patients with BD. They are particularly helpful to clients who are unfamiliar with the mental health system and newly developed interventions, such as peer support services. Finally, the present project is one of the few studies in non-Western countries to adopt a partnership between clients and academic researchers in the mental health field. Accessing clients’ expert-by-experience represents an important step in recognizing their contribution to the co-construction of knowledge [37, 51]. We are hopeful that the present project will encourage the creative incorporation of PSWs into psychiatric research, into the conceptualization, design, implementation, data interpretation, and dissemination of results, and thereby facilitate improvements in service provision in a resource-limited health sector [58, 59].

## Data Availability

Supporting data will not be made available as they contain indirect identifiers and releasing them could breach clients’ confidentiality.

## Abbreviations

BD: Bipolar disorder
ICCMW: Integrated Community Centre for Mental Wellness
NGO: Non-governmental organization
